# Enhanced External Counterpulsation as a Novel Treatment for Post-acute COVID-19 Sequelae

**DOI:** 10.7759/cureus.14358

**Published:** 2021-04-07

**Authors:** Joshua Kyle Dayrit, Monica Verduzco-Gutierrez, Ann Teal, Sachin A Shah

**Affiliations:** 1 Department of Pharmacy, University of the Pacific, Stockton, USA; 2 Department of Rehabilitation Medicine, Joe R. and Teresa Lozano Long School of Medicine at University of Texas Health San Antonio, San Antonio, USA; 3 Medical Affairs, Flow Therapy, Fort Worth, USA

**Keywords:** covid-19, enhanced external counterpulsation, eecp, long covid, pasc

## Abstract

Severe acute respiratory syndrome coronavirus 2 (SARS-CoV-2) is the virus responsible for the coronavirus disease 2019 (COVID-19) pandemic. As patients recover from COVID-19, some continue to report persisting symptoms weeks to months after acute infection. These effects have been referred to as post-acute sequelae of SARS-CoV-2 infection (PASC). We report the case of a 38-year-old woman suffering from PASC symptoms following acute COVID-19 in October 2020. During her acute infection phase, she had a home recovery and reported her predominant symptoms as fatigue, headaches, body pain, and shortness of breath. After most of her symptoms were resolved, she continued to have periodic episodes of fatigue and headaches, along with random shortness of breath while at rest and during activities for months beyond the acute phase of the illness. She also noted the presence of “brain fog,” as if lacking the same clarity that she had prior to her illness. These symptoms persisted for three months before the patient underwent enhanced external counterpulsation (EECP) therapy in one-hour sessions, three times per week. This therapy was chosen based on the mechanism of action of EECP benefiting patients with ischemic cardiovascular diseases. After one week, her “brain fog” had improved, with shortness of breath improving after 1.5 weeks. The patient reported returning to pre-COVID health and fitness after approximately five weeks of EECP treatment. To our knowledge, this is the first case of using EECP for post-COVID shortness of breath, fatigue, and “brain fog.”

## Introduction

In the unprecedented times of the coronavirus disease 2019 (COVID-19) pandemic, there are increasing reports of persistent symptoms weeks to months after recovery from acute COVID-19 [1]. This is a case of long-term effects following acute COVID-19, characterized by fatigue, dyspnea, and “brain fog” (an inability or difficulty in concentrating or focusing). Many people in this population have considered themselves as “COVID-19 long haulers” or suffer from “long COVID.” Recently, the National Institutes of Health have named the condition as post-acute sequelae of SARS-CoV-2 infection (PASC). Here, we present a case of PASC that was treated with enhanced external counterpulsation (EECP).

## Case presentation

In October 2020, a 38-year-old Hispanic woman with no preexisting health conditions noted the onset of what she was certain to be her usual allergy symptoms, including itchy eyes, sneezing, sinus drainage, and headache. She was generally healthy and was aware of standard COVID19 precautions being a working healthcare professional. As a precautionary measure she took an additional temperature measurement, which came to be normal. Overnight, she noticed a new onset of fever, severe body aches, and pronounced fatigue. The next day she was tested for both influenza A/B and COVID-19 via antigen test, with a negative flu result but a positive COVID-19 result. For verification, she also took a COVID-19 polymerase chain reaction test that also resulted in a positive for infection. She was prescribed conventional therapy at the time, including a cocktail of three vitamins (vitamin D-3 2,000 IU daily, zinc 50 mg daily, and vitamin C 500 mg daily), hydroxychloroquine 200 mg twice daily for six days, and Tylenol 500 mg two tablets every four to six hours as needed. She had an at-home recovery and reported her predominant symptoms during the acute phase as fatigue, headaches, body pain, and shortness of breath.

After most of her symptoms were resolved, she continued to have periodic episodes of fatigue and headaches, along with random shortness of breath while at rest and during activities for months beyond the acute phase of the illness. She also noted the presence of “brain fog,” as if lacking the same clarity that she had prior to her illness. These symptoms persisted for three months before the patient underwent EECP for one-hour sessions, three times per week. The patient underwent a non-standard EECP regimen of three days per week for five weeks due to convenience. This therapy was chosen based on EECP’s unique mechanism of action and the known cardiac and extra-cardiac effects. After one week, her “brain fog” had improved, with shortness of breath improving after 1.5 weeks. The patient reported returning to pre-COVID-19 health and fitness after approximately five weeks of EECP treatment.

## Discussion

Several population studies in post-COVID-19 patients have emerged around the world, ranging in follow-up time from a few weeks to several months. An early study conducted during March-June 2020 by the United States Centers for Disease Control and Prevention followed 274 patients at two to three weeks after initial positive COVID-19 test. They found that 34% of the patients reported at least one symptom, the most common symptoms included: cough (43%), fatigue (35%) congestion (30%), dyspnea (29%), confusion (20%) and chest pain (20%) [1]. A prospective cohort study of 1,733 patients in Wuhan, China evaluated patients at six months from symptom onset. The study found that 76% of the patients reported at least one symptom at follow-up, most commonly: fatigue/muscular weakness (76%), dyspnea (26%), sleep difficulties (26%), and anxiety/depression (23%) [2].

The underlying pathophysiology of PASC is still being understood. A leading hypothesis is that SARS-CoV-2 causes endothelial dysfunction by infecting endothelial cells around the body via the angiotensin converting enzyme-2 receptor, prompting release of inflammatory cytokines and compromising the epithelial-endothelial barrier at late stages of infection. Several studies assessing post-acute sequelae have found changes in cardiovascular, pulmonary, and neuropsychiatric organ systems. Puntmann et al. performed cardiac magnetic resonance imaging in 100 patients approximately 71 days after the initial COVID-19 diagnosis and found abnormal findings in 78% of the patients [3]. Huang et al. assessed pulmonary function in 57 patients at one month after discharge, finding diffusing capacity for carbon monoxide (DLCO) below 80% of predicted in 53% of the patients, with higher incidences in patients with severe infection compared to non-severe infection. Additionally, patients with severe COVID-19 had a mean 56 m shorter six-minute walking distance compared to non-severe patients [4]. Zhou et al. assessed cognitive function and inflammatory markers in 29 patients two to three weeks after infection, finding significant decreases in cognitive function in the sustained attention domain along with significant elevations in c-reactive protein [5]. Another study performed brain MRI in 51 COVID-19 patients three months after discharge, finding decreases in cerebral blood flow and changes to white and gray matter in patients with severe infection [6]. These studies show the extent of severity we may see in PASC patients.

Based on the pathophysiological changes with PASC, EECP could be a suitable treatment method for such patients. EECP is a Food and Drug Administration-approved, non-invasive outpatient therapy for patients with chronic stable angina and/or heart failure of ischemic etiology. Standard therapy involves 35 one-hour treatments over seven weeks. Patients lie on a treatment table where compressive cuffs (similar to large blood pressure cuffs) are securely wrapped around their calves, thighs, and buttocks. These cuffs inflate in a distal-to-proximal sequence during diastole and deflate simultaneously just prior to the onset of systole. Inflation and deflation are specifically timed to the patient’s electrocardiogram to optimize therapeutic benefit. This mechanical compression induces physiologic and biochemical changes in the vasculature, leading to angiogenesis and increased coronary function. The compression produces pulsatile shear stress, which has been associated with improvements in systemic endothelial function via modulation of vasodilating (e.g., nitric oxide) and pro-inflammatory agents (e.g., tumor necrosis factor alpha) [7].

In patients with underlying cardiovascular disease, EECP has been shown to significantly improve Canadian Cardiovascular Society angina class, Duke Activity Score Index, Seattle Angina Questionnaire, and six-minute walking distance, all of which are independent markers associated with mortality benefits. EECP has also been shown to improve time to ST-segment depression, treadmill exercise duration, ejection fraction, and cardiac output/efficiency [7].

The benefits of EECP are also evident in patients without underlying cardiovascular disease. A recently published study showed improved exercise endurance in those with low-to-normal exercise endurance, as well as in chronic obstructive pulmonary disease patients. Significant improvements in maximum oxygen uptake (VO_2_-MaxKg) were seen after three weeks of EECP therapy [8]. Lastly, EECP has also been known to enhance cerebral blood flow, collateralization in the ischemic regions of the brain, and cognitive function. All these factors, in addition to improved endothelial function, contribute to the mechanisms by which EECP may provide relief in patients with fatigue, dyspnea, chest pain, and “brain fog” symptoms.

## Conclusions

To our knowledge, this is the first case of using EECP for post-COVID dyspnea, fatigue, and “brain fog.” There are no current guidelines for the treatment of PASC, and management of the condition is largely based on patient presentation and symptoms and requires the efforts of a multidisciplinary team. This initial case report of a patient recovering from her post-acute COVID-19 symptoms suggests EECP may serve as a novel treatment modality for the management of PASC. More data are needed to further validate these findings.

## References

[1] Tenforde MW, Kim SS, Lindsell CJ (2020). Symptom duration and risk factors for delayed return to usual health among outpatients with COVID-19 in a multistate health care systems network - United States, March-June 2020. MMWR Morb Mortal Wkly Rep.

[2] Huang C, Huang L, Wang Y (2021). 6-month consequences of COVID-19 in patients discharged from hospital: a cohort study. Lancet.

[3] Puntmann VO, Carerj ML, Wieters I (2020). Outcomes of cardiovascular magnetic resonance imaging in patients recently recovered from coronavirus disease 2019 (COVID-19). JAMA Cardiol.

[4] Huang Y, Tan C, Wu J (2020). Impact of coronavirus disease 2019 on pulmonary function in early convalescence phase. Respir Res.

[5] Zhou H, Lu S, Chen J (2020). The landscape of cognitive function in recovered COVID-19 patients. J Psychiatr Res.

[6] Qin Y, Wu J, Chen T (2021). Long-term micro-structure and cerebral blood flow changes in patients recovered from COVID-19 without neurological manifestations [Online ahead of print]. J Clin Invest.

[7] Raza A, Steinberg K, Tartaglia J, Frishman WH, Gupta T (2017). Enhanced external counterpulsation therapy: past, present, and future. Cardiol Rev.

[8] Zhao M, Huang Y, Li L (2020). Enhanced external counterpulsation efficacy on exercise endurance in COPD patients and healthy subjects: a pilot randomized clinical trial. Int J Chron Obstruct Pulmon Dis.

